# Guardians and Operation Fees

**Published:** 1897-11-20

**Authors:** 


### EDITOR'S LETTER-BOX.

[Our Correspondents are reminded that prolixity is a great bar to publication, and that brevity of style and conciseness of statement greatly facilitate early insertion.]

### GUARDIANS AND OPERATION FEES.

Dr. Woodley Stocker asks our opinion under the following circumstances. It appears that he is Poor Law medical officer for the parish of Willesden, and also medical officer to the cottage hospital there. Willesden has not yet been provided with a Poor Law infirmary, and Dr. Stocker having two patients, one of whom required amputation of the breast and the other amputation of the leg, he had them taken to the cottage hospital, where he did the necessary operations, charging the guardians the sum of £10, which is the scheduled fee according to his contract with them. Payment, however, was refused on the ground that the auditor would not allow it because the operations were done at the cottage hospital. On the matter being referred to the Local Government Board, however, the reply was that they would allow the fee. Some of the guardians, however, still object to its being paid, and we are asked our opinion. To this we would answer that the Local Government Board allows the charge, and they are right according to precedent and practice. The late Albert Napper, the founder of cottage hospitals, fought this battle ior the profession and won it thirty years ago, as shown in "Cottage Hospitals," 3rd edition, pages 50 and 51, where the subject is fully dealt with. In regard to the statement that the guardians have now subscribed £10 to St. Mary's Hospital to take all operations for the parish we can only say that if the statement is correct it displays a condition of affairs which is very improper indeed. The guardians are responsible for the proper treatment of their sick, and any attempt to transfer this responsibility to institutions supported by voluntary contributions and officered by an honorary staff is a mere endeavour to save the rates at the expense of the subscriptions of the charitable; and, we may add, any hospital which allows itself to be a participator in such a transaction is misappropriating the funds entrusted to it.

